# Structure and mechanisms of viral transcription factors in archaea

**DOI:** 10.1007/s00792-017-0951-1

**Published:** 2017-07-05

**Authors:** Carol Sheppard, Finn Werner

**Affiliations:** Division of Biosciences, Institute of Structural and Molecular Biology, University College London, London, WC1E 6BT UK

**Keywords:** Transcription regulator, RNA polymerase, Archaea, Virus, Evolution

## Abstract

Virus-encoded transcription factors have been pivotal in exploring the molecular mechanisms and regulation of gene expression in bacteria and eukaryotes since the birth of molecular biology, while our understanding of viral transcription in archaea is still in its infancy. Archaeal viruses do not encode their own RNA polymerases (RNAPs) and are consequently entirely dependent on their hosts for gene expression; this is fundamentally different from many bacteriophages and requires alternative regulatory strategies. Archaeal viruses wield a repertoire of proteins to expropriate the host transcription machinery to their own benefit. In this short review we summarise our current understanding of gene-specific and global mechanisms that viruses employ to chiefly downregulate host transcription and enable the efficient and temporal expression of the viral transcriptome. Most of the experimentally characterised archaeo-viral transcription regulators possess either ribbon–helix–helix or Zn-finger motifs that allow them to engage with the DNA in a sequence-specific manner, altering the expression of a specific subset of genes. Recently a novel type of regulator was reported that directly binds to the RNAP and shuts down transcription of both host and viral genes in a global fashion.

## Introduction: archaeal viruses and transcription regulation

Although viruses are not classified as living organisms per se, they represent the most abundant biological entities on the planet and can inhabit some of the biosphere’s most extreme environments. Viruses infect all three domains of life and are believed to have origins deeply rooted in life’s phylogenetic tree (Villarreal and Witzany 2010; Forterre 2006). They play a major role in horizontal gene transfer and therefore provide a key driving force in evolution (Koonin and Wolf 2012; Forterre and Prangishvili 2013). An excess of 6000 bacteriophage (phage)—viruses infecting bacteria have been discovered to date, of which the vast majority possess the icosahedral head–tail-like structure which is classically associated with the phage (Ackermann and Prangishvili 2012). The first archaeal virus was isolated from a halophilic *Euryarchaeon* and was originally mistakenly classified as a phage owing to its head–tail morphology and the fact that the archaea had not yet been recognised as a separate domain of life (Torsvik and Dundas 1974). Considering the late assignment of the archaeal kingdom it is not surprising that only a small number of archaea infecting viruses have been identified. In recent years, there has been a surge in the identification and study of archaeal viruses and although still relatively few in number, approximately 160, they already display an unprecedented morphological diversity of which most have never been observed before. In addition to the head–tail structures originally discovered, the repertoire of shapes has rapidly expanded and now includes rods, flexuous filaments, droplets, spindles, spindles with single/double tails and even bottle shaped particles (reviewed in Pietila et al. 2014). All the archaeal viruses isolated to date belong to either one of the two major archaeal phyla; the *Crenarchaeota* and the *Euryarchaeota*. The distinct morphology of the archaeal viruses forms the major criteria for their taxonomical ranking into 16 different families; *Myoviridae*, *Pleolipoviridae*, *Podoviridae*, *Siphoviridae* and *Sphaerolipoviridae* which infect the *Euryarchaea*, *Ampullaviridae*, *Bicaudaviridae*, *Clavaviridae*, *Fuselloviridae*, *Globuloviridae*, *Guttaviridae*, *Lipothrixviridae*, *Rudiviridae*, *Spiraviridae*, *Turriviridae*, (Pietila et al. 2014; Prangishvili 2013; Pina et al. 2011) and the most recent *Tristromaviridae* (Rensen et al. 2016) which infect the *Crenarchaea*.

Despite their heterogeneous morphotypes, all archaeal viruses possess DNA genomes. With the exception of just a few single-stranded DNA viruses (Pietila et al. 2009; Sencilo et al. 2012; Mochizuki et al. 2012) almost all are double stranded, and no viruses with RNA genomes have been found yet. Archaeal viruses represent an immense resource of genetic diversity; the vast majority of the viral ORFs share little sequence homology with genes of known function including genes from their hosts and even of other viruses from the same family (Dellas et al. 2013; Iranzo et al. 2016). This limited sequence similarity curbs the usefulness of bioinformatics approaches and makes it challenging to make predictions based on protein structure and function. Surprisingly, genomic analyses of archaeal viruses have revealed a distinct lack of RNA polymerases suggestive of an explicit reliance on the host replication and transcription apparatuses (Prangishvili et al. 2006). A number of viral ORFs have predicted functions as transcription regulators that indubitably have to act on the host RNAP system. Despite low sequence conservation, structural prediction software has suggested the presence of structural folds including helix–turn–helix (HTH) and zinc (Zn)-finger motifs that are characteristic of the DNA-binding domains of transcription factors. However, only a small number of these putative transcription factors have actually been functionally characterised and they operate via canonical promoter occlusion mechanisms. Recently a completely novel type of regulator has been discovered and its molecular mechanisms characterised in great detail. This factor is encoded by the *Acidianus* two-tailed virus and directly targets and binds to the host RNAP, inactivates it in a reversible fashion using an allosteric mechanism. Here we report our current understanding of archaeo-viral transcription regulators and how they are employed by archaeal viruses to modulate the host and virus gene expression programmes. We provide details on their structures and discuss their mechanisms of action in the context of the life cycle of the virus.

## The archaeal transcription machinery

Archaea, like bacteria, are bona fide prokaryotes; however, the archaeal transcription machinery portrays a streamlined version of the eukaryotic RNAP systems (Werner and Grohmann 2011). Compared to their eukaryotic counterparts, archaeal model systems are advantageous because of their high experimental tractability. Hyperthermophilic archaeal factors are generally smaller, without ‘floppy’ N- and C-termini and therefore they make stable and soluble recombinant proteins ideally suited for structure determination. Archaeal RNAPs are amenable to in vitro reconstitution from individual subunits, allowing their perturbation by mutagenesis and the incorporation of molecular probes including fluorescent dyes (Schulz et al. 2015; Werner and Weinzierl 2002). Furthermore, archaea possess relatively small genomes (1–5 Mb) that enable comprehensive yet detailed system analyses combining whole genome occupancy, transcription start site (TSS)-mapping and transcriptomic analyses (Smollett et al. 2017). For the aforementioned reasons, archaea represent ideal model systems for the study of gene expression.

The archaeal RNAP is structurally and functionally similar to the eukaryotic RNA polymerase II (Hirata et al. 2008; Korkhin et al. 2009) and shares the minimal requirements for general initiation factors including the TATA-binding protein (TBP) and transcription factor B (TFB) which are homologous to TBP and TFIIB in eukaryotes, respectively (Hausner et al. 1996; Qureshi et al. 1997). TBP and TFB interact with TATA and BRE sequence motifs upstream of the transcription start site and recruit the RNAP to the promoter to form the pre-initiation complex (PIC), whereby the DNA initially remains in its duplex or ‘closed’ form (Hausner et al. 1996). Subsequent localised melting of the DNA around the transcription start site and the loading of the DNA template strand into the RNAP active site denote the formation of the ‘open’ complex and occur concurrently to large-scale conformational changes within the RNAP (He et al. 2013; Chakraborty et al. 2012). The transition from the ‘closed’ to the ‘open’ complex is stimulated by the third basal factor TFE that is homologous to TFIIE in eukaryotes (Bell et al. 2001; Blombach et al. 2015).

In contrast to the parallels drawn between the archaeal and eukaryotic basal transcription machinery many of the auxiliary proteins involved in the regulation and fine-tuning of transcription in archaea are reminiscent of those in bacteria (Bell 2005; Kyrpides and Ouzounis 1999). The majority of characterised archaeal transcription factors are repressors and function via promoter occlusion. Some of the most well-studied examples include Lrs14 (Napoli et al. 1999; Bell and Jackson 2000), TrmB (Lee et al. 2003), Mdr1 (Bell et al. 1999), LrpA (Brinkman et al. 2000; Dahlke and Thomm 2002), PhrA (Vierke et al. 2003) and NrpR (Lie and Leigh 2003) all of which act either by interacting with the DNA and occluding the binding of TBP and/or TFB or by preventing the recruitment of RNAP. Well-studied transcription activators include Ptr2 and TFB-RF1 which stimulate transcription by enhancing the recruitment of TBP and TFB to the promoter and as such follow the eukaryotic paradigm of transcription regulation (Ouhammouch et al. 2003; Ouhammouch et al. 2005; Ochs et al. 2012). All structurally characterised archaeal transcription factors include a 2 α-helical structural fold termed a helix–turn–helix (HTH) motif that is particularly prevalent amongst prokaryotes. The second helix, known as the recognition helix is the main contributor to the interaction surface, inserting itself into the major groove of the DNA. It forms hydrogen bonds and van der Waals interactions between the side chains of the protein and exposed bases of the DNA thereby providing the sequence specificity of the regulator (Brennan and Matthews 1989; Aravind et al. 2005).

The catalytic site of all multisubunit RNAPs is formed at the interface between two double-psi beta barrels (DPBB) residing in the two large subunits. The DPBB of the largest subunit (Rpo1 in archaea) harbours three aspartic acid residues located within the NADFDGD sequence motif that chelate the catalytic Magnesium ions A and B in the active centre of the enzyme (Sosunov et al. 2005). Extensive sequence searches of archaeo-viral genomes have failed to identify these signature motifs (Sheppard et al. 2016) and it seems highly unlikely that the archaeal viruses encode their own RNAP, but rather rely on the host enzyme. In support of this notion, promoter motif analysis of archaeo-viral genomes has revealed clear TATA and BRE elements indicating that they are likely to exploit the host TBP and TFB initiation factors (Reiter et al. 1988; Kessler et al. 2004). In addition to the canonical motifs, in SIRV1 and SIRV2 the majority of promoter regions include a GTC motif that is located downstream of the TATA box and is proposed to act as a virus-specific *cis*-regulatory element (Kessler et al. 2004). The absence of viral RNAPs and the presence of archaeal promoter elements implies that the archaeal viruses are completely dependent on their host transcription machinery for the expression of their own genomes. Consequently, the archaeal transcription machinery is not only subject to refinement by archaeal transcription factors but also by modulation from the competing archeao-viral encoded factors. The question remains as to how the viruses are able to re-route the archaeal RNAP away from the transcription of the host genes and towards the expression of specific viral genes in an efficient and timely manner. Below we detail our current knowledge of archaeo-viral transcription regulators that have been identified and functionally characterised at the molecular level. Transcription regulators can be divided into two categories; (1) those that regulate transcription in a gene-specific fashion by interacting with the DNA, and (2) transcription factors that modulate gene expression globally by interacting with the RNAP.

## Transcription regulation by DNA-binding factors

The SvtR, AvtR, F55, C68 and p106 transcription factors regulate transcription in a gene-specific manner by interacting with specific DNA sequence motifs. The putative consensus-binding sequences for SvtR (ATnnTTCAAnAnnnnnAAAATG) and AvtR (ATnnTnnTAnnACnTT) form imperfect inverted repeats whereas F55 interacts with sequences that contain two tandem repeat sequences with an ATAGATAGAGT consensus and C68 interacts recognises the TATG(C/GG)TTTTC motif.

**Fig. 1:**
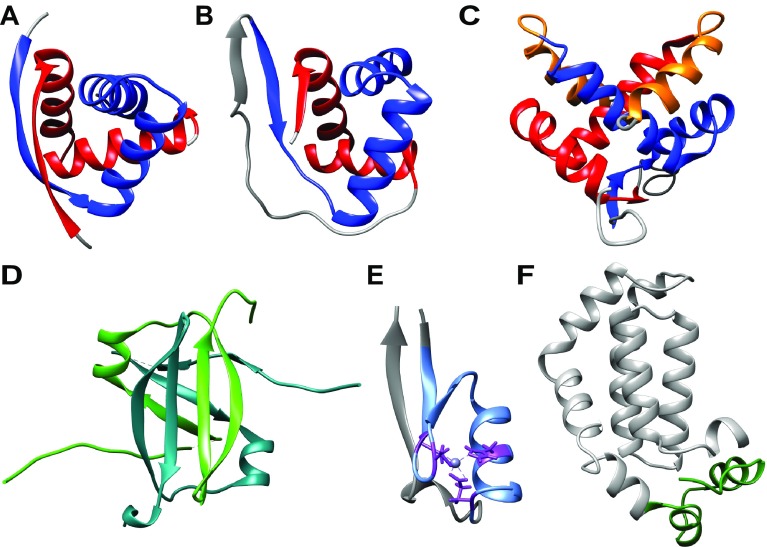
Structural features of transcription regulators from archaeal viruses. **a** Dimer of AvtR (pdb 4hv0) with each RHH motif-containing monomer highlighted in *red* and *blue*, respectively. **b** SvtR (pdb 2kel) encompassing two fused RHH motifs, each highlighted in *red* and *blue*, respectively. **c** Dimer of E73 (pdb 4aai) with the RHH motif of one monomer highlighted *red* and the *second blue*. The 3rd α-helix, conferring the RH3 fold is coloured in *orange* for both monomers. **d** C68 (pdb 3o27) with monomers coloured in *cyan* and *green*, respectively. **e** AFV1p06 (pdb 2lvh) with the Zn-finger motif highlighted in *light blue* and the Zn-coordinating cysteine residues shown in *stick representation* and coloured in *purple*. **f** Homology model of ATV RIP with the functionally important tail motif highlighted in *dark green*

**Fig. 2:**
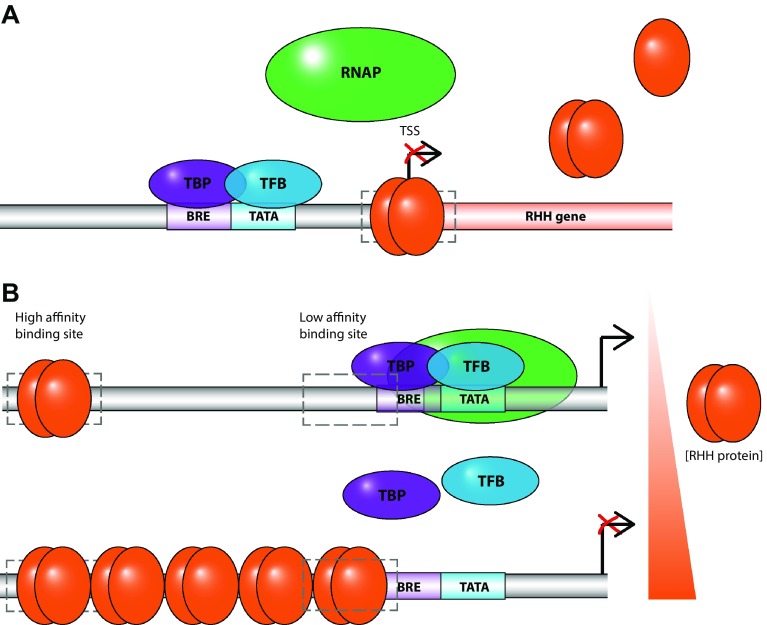
RHH transcription regulators repress transcription by a promoter occlusion mechanism. **a** RHH proteins bind to recognition motifs that often overlap with the transcription start site (TSS) or promoter elements of their own genes, and thereby prevent the recruitment of the transcription preinitiation complex (PIC). **b** RHH proteins tend to preferentially bind to high affinity sites located distal to the promoter region. At higher regulator concentrations they begin to oligomerise along the DNA towards the promoter elements (BRE and TATA), which leads to the occupation of degenerate (low affinity) binding sites and interference with the assembly of the PIC

SvtR, AvtR, F55 and E73 possess a variation of the HTH motif known as the ribbon–helix–helix (RHH) fold (Fig. 1). RHH proteins are absent in the eukaryotic domain but are common amongst transcription regulators in bacteria, archaea and phage. The RHH motif consists of a N-terminal β-strand followed by 2 α-helices. The RHH domain is formed by the dimerisation of two monomers, which each contribute a β-strand to form an anti-parallel β-sheet that makes sequence-specific contacts with the major groove of the DNA. RHH proteins typically comprise hydrophobic residues at residues 3, 5 and 7 of the β-sheet which are important for making contact with the adjacent DNA nucleotide bases (Prangishvili et al. 2006). Furthermore, RHH dimers bind to multiple operator sites in a cooperative manner to form higher order oligomers along the DNA (Schreiter and Drennan 2007) (Fig. 2).

### *Sulfolobus* virus transcription factor, SvtR

The *Sulfolobus* virus transcription regulator (SvtR, gp08) is a small, 6.6 kDa protein encoded by *Sulfolobus islandicus* rod-shaped virus 1 (SIRV1). SvtR consists of a RHH fold and forms a homodimer (Fig. 1a) which is highly reminiscent of bacterial RHH proteins including CopG (plasmid copy number regulator), NikR (nickel responsive element) and MetJ (methionine repressor) which all act as transcriptional repressors. At the sequence level, SvtR shares limited similarity with these proteins with the exception of key hydrophobic residues located in the interface between the monomers, highlighting the importance of the dimerisation. Like bacterial RHH proteins, SvtR is able to bind to its own (*gp08*) promoter where it acts as a repressor of transcription and thus downregulates its own expression. SvtR is known to bind to three other sites on the viral genome of which it has the highest apparent affinity for the promoter region of the *gp30* gene that encodes a structural protein involved in the formation of tail fibres which are required for virion assembly (Guilliere et al. 2009). The primary role of SvtR may be to prevent the premature expression of gp30 until it is required during the later stages of virus development. SvtR is only able to repress transcription under conditions where the levels of TBP and TFB are limiting, which would imply that SvtR functions by competing with their recruitment to TATA and BRE motifs and possibly occluding their binding altogether. However, both TATA and BRE sequences in the *gp30* promoter are located approximately 80 nucleotides downstream from the SvtR-binding site. A possible explanation for this behaviour is that binding of SvtR to the upstream high affinity binding site triggers the polymerisation of SvtR along the DNA into the promoter region containing the TATA and BRE sequences, as is described of bacterial RHH transcription factors (Schreiter and Drennan 2007).

### *Acidianus* virus transcription factor, AvtR

The *Acidianus* virus transcriptional regulator (AvtR) is a 11.9 kDa protein encoded by *Acidianus* filamentous virus 6 (AFV6) and is highly conserved in the Beta-lipothrixvirus genus. Unlike other RHH proteins which need to form dimers to interact with the DNA, AvtR interacts with DNA as a monomer. The AvtR monomer in turn is composed of two tandem RHH motifs that are connected by a linker (Fig. 1b). The 2.6 Å resolution crystal structure of AvtR can be superimposed onto dimers of other RHH proteins and like SvtR, it shows high structural homology to the bacterial plasmid copy number regulator, CopG. AvtR binds to eight potential sites in the AFV6 genome, which are all conserved in homologous genes of the same genus. Like other RHH transcription factors AvtR represses the expression of its own gene (*gp29*), and in addition is able to both activate and repress transcription from the *gp30* gene in a concentration-dependent manner. Intriguingly *gp30* is predicted to encode a further RHH protein. This makes AvtR the only known archaeo-viral protein to act as both a repressor and an activator of transcription. DNaseI footprinting assays showed that AvtR protects a stretch of about 100 nucleotides between the *gp29* and *gp30* promoters which is in support of the hypothesis that several subunits are able to oligomerise along the DNA as also suggested for SvtR. It is proposed that AvtR first binds to high affinity operator sites and then binds with weaker affinity to more degenerate sites. The stimulatory effect of AvtR is only observed at low protein concentrations and diminishes as AvtR concentration increases and oligomerises along the DNA. Therefore, the expression levels of AvtR may serve as a tipping point between stimulation and repression of targeted genes (Peixeiro et al. 2013).

### *Sulfolobus* spindle-shaped virus transcription factor, F55

F55 is a 6.3 kDa protein encoded by *Sulfolobus* spindle-shaped virus 1 (SSV1) of the *Fuselloviridae* family and is probably the archaeo-viral transcription factor with the most well defined biological role. SSV1 is a lysogenic virus that is unique among *Crenarchaeal* viruses in its propensity to enter a replicative life cycle upon exposure to UV light (Martin et al. 1984). Like the bacterial phage lambda, SSV1 transcribes its genome in a temporal manner and its early UV inducible genes are clustered together in a small region (Frols et al. 2007; Hendrix 1983). F55, is reminiscent of the lambda CI repressor protein as it too is responsible for the maintenance of the lysogenic state by preventing the expression of the early genes (Gilbert and Muller-Hill 1966; Fusco et al. 2013, 2015).

F55 contains a RHH domain that shares approximately 50% sequence homology with other RHH transcription regulators. F55 is dimeric and binds in a concentration-dependent manner to tandem repeat sequences—ATAGATAG—within or adjacent to the promoter regions of the immediate-early genes T_5_, T_6_, T_ind_ as well as the promoter of its own gene T_lys_. The upstream sequences of T_5_, T_6_, T_ind_ contain two binding sites for F55 each containing two tandem repeats, whereas the promoter region of T_lys_ only contains one binding site. F55 is highly expressed during the lysogenic state where it binds to sites that overlap with the TSS and BRE element to repress transcription from its target genes (Fusco et al. 2013). Two hours post UV irradiation, F55 levels are depleted by half leading to its dissociation from the lower affinity sites in the T_ind_ and T_lys_ promoters. A further decrease in F55 levels after 4 h post UV irradiation also relieves the repression from the higher affinity sites within the promoters of the T_5_ and T_6_ genes. It is not known precisely what mechanism causes the initial depletion of F55 and although it seems that there is a certain degree of post-transcriptional degradation it may be inferred from the comparisons with the phage lambda system that F55 protein could be degraded as part of the hijacked host SOS response (Sauer et al. 1982). Overall, this behaviour suggests that F55 acts as a molecular switch for the transition from the carrier state to the expression of the early viral genes and eventually viral replication (Fusco et al. 2015).

### *Sulfolobus* spindle-shaped virus factor E73

E73 is encoded by *Sulfolobus* spindle-shaped virus-Ragged Hills (SSV-RH) with orthologs identified in at least six other SSV genomes including E51, the product of the SSV1 early T_5_ transcript (Frols et al. 2007). E73 is a 73 aa homodimeric protein comprising a recognisable RHH fold that is distinctive due to the presence of a tightly integrated 3rd C-terminal α-helix (Schlenker et al. 2009) (Fig. 1c). This unique structure, termed the RH3 fold allows the α_2_ and α_3_ residues of one monomer to encompass the α_2_ of the adjoining monomer. The additional α-helix is likely to contribute to the extreme thermostability of the dimer. Furthermore, this adaptation also results in the creation of a positively charged cleft at the opposing side to the antiparallel β-sheet, which is surmised to form a ligand binding site (Schlenker et al. 2012). The core RHH motif of E73 shares high structural similarity with the bacterial transcription repressors PutA (proline utilisation protein), CopG and the Arc repressor protein as well as the archaeo-viral repressor SvtR. Therefore, by extension E73 is likely to also function as a repressor, however EMSA experiments have only shown E73 to interact with SSV-RH DNA in an apparent non-specific manner (Schlenker et al. 2012).

### *Sulfolobus* plasmid–virus transcription factor, C68

pSSVx is a hybrid genetic element that shares similarities with both plasmids and *Fuselloviruses* (Arnold et al. 1999). pSSVx infects/transforms various *Sulfolobales* and encodes nine ORFs, with *orfc68* oriented in the reverse orientation to the remaining eight genes. *Orfc68* encodes a 7.7 kDa protein, C68, that only shares homology with cellular *Crenarchaeal* factors and *Crenarchaeal* plasmid-encoded proteins of unknown function (Contursi et al. 2011). C68 proteins employ an unusual swapped hairpin mechanism for dimerisation that is characterised by a β-sheet scaffold (Fig. 1d). C68 shares structural similarity to the bacterial AbrB, Abh, SpoVT and MazE proteins, all of which are transcription factors that include an N-terminal swapped hairpin fold responsible for DNA binding and in some cases a C-terminal domain (not present in C68) for oligomerization (Coles et al. 2005). C68 binds to the intergenic region between its own gene promoter and the divergently orientated *orf60* promoter which encodes a CopG homologue, but it remains unknown how C68 affects their activity. C68 is recruited to two binding sites via the TATG(C/GG)TTTTC consensus motif, a high affinity site that overlaps with the BRE motif of the *orfc68* promoter and a lower affinity site located equidistant between the promoters of *orf68* and *orf60*. The recognition motif of C68 is also present in the promoter of a predicted DNA-binding protein located in the CRISPR locus of *S. islandicus*, and thus it has been speculated that C68 could be involved in the regulation of the host immune response (Contursi et al. 2011).

### *Acidianus* filamentous virus 1 factor, p06

The zinc-finger motif is a common nucleic acid-binding domain in eukaryotic transcription factors but is extremely rare in bacteria. Only one archaeal Zn-finger-containing protein has been functionally characterised, AFV1p06 (ORF59a). This 7 kDa protein is encoded by the *Acidianus* filamentous virus 1 (AFV1) and has homologues in the *Crenarchaeal* spindle-shaped viruses. Interestingly AFV1p06 is related to ORFs encoded by both *Cren*- and *Euryarchaeaota*, which are believed to be derivatives of the viral factor judging from their flanking viral att-like sites, hallmarks of site-specific recombination. AFV106 contains an atypical Zn-finger motif with two cysteines, one histidine and one glutamic acid residue that chelate the Zn ion (Fig. 1e). AFV1p06 binds DNA with a preference for GC-rich sequences but currently no specific recognition motif or target genes have been identified (Guilliere et al. 2013).

## Transcription regulation by RNA polymerase-binding factors

### *Acidianus* two-tailed virus RNAP inhibitory protein, RIP

**Fig. 3:**
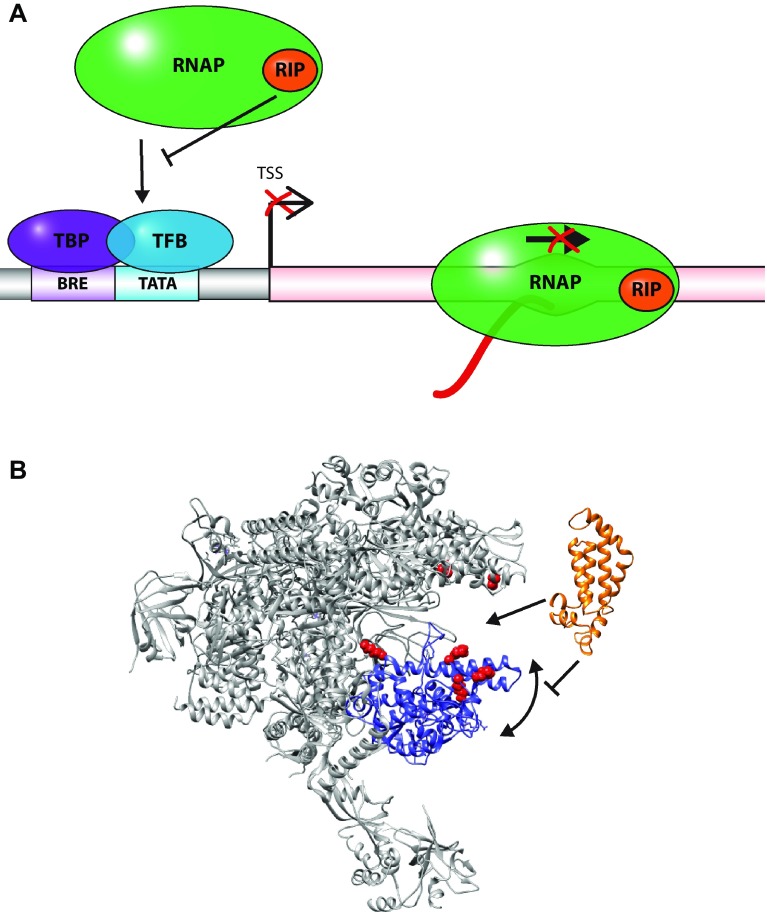
Global regulators of transcription interact with directly with RNAP. **a** Regulatory factors that interact with the RNAP and not with DNA recognition motifs affect transcription independent of the promoter context. RNAP-binding regulators can interfere with PIC assembly, the catalytic mechanisms of RNAP, either during initiation or elongation of transcription. **b** Despite being abundant in bacteriophages, the ATV-encoded RIP (RNAP inhibitory protein), shown in *orange*, is the only example of a global regulator that has been characterised in archaea or archaeal viruses so far. RIP is inserted into the DNA-binding channel of RNAP as shown by chemical crosslinking to RNAP (cross linked residues highlighted as *red spheres*). Upon binding, RIP conformationally locks the RNAP clamp (highlighted in *blue*) which destabilises the PIC and inhibits transcription at the level of initiation, and also interferes with catalysis during transcription elongation, thus effectively repressing transcription on a global level

All of the archaeo-viral transcription factors described above regulate transcription by binding to the DNA in a sequence-specific fashion. Recently the molecular mechanisms of a novel type of global transcription regulator were characterised in great detail. The RNAP inhibitory protein, or RIP, exerts its effects by interacting directly with the RNAP and independently of the template DNA context. RIP (ORF145) is a 16.8 kDa protein encoded by the *Acidianus* two-tailed virus (ATV) that binds apical to, or even partially inserted into, the DNA-binding channel of the RNAP with nanomolar affinity. One of the key target sites is the RNAP clamp, a domain renowned for its flexibility that enables the widening (‘opening’) or narrowing (‘closing’) of the DNA-binding channel that is required for the productive progression of RNAP through the transcription cycle (Schulz et al. 2016). RIP binding to RNAP impairs the movement of the clamp and locks it in an intermediate conformation that (1) prevents the RNAP from forming stable pre-initiation complexes and (2) renders the RNAP catalytically inert (Sheppard et al. 2016) (Fig. 3). Notably the clamp domain from bacterial, archaeal and eukaryotic RNAPs serves as a regulatory ‘hot spot’ for several transcription factors including sigma-70, TFE and TFIIE that stimulate the melting of the DNA during ‘open’ complex formation, Spt4/5, NusG and RfaH that modulate the processivity and termination of transcription (Belogurov et al. 2009; Grohmann et al. 2011; Plaschka et al. 2016; Werner 2012). In the context of the PIC, the binding of TFE to RNAP triggers clamp opening, increases the apparent affinity of RIP for RNAP and enhances its inhibitory effects of RIP on PIC formation and transcription (Sheppard et al. 2016; Schulz et al. 2016). In addition to its inhibitory activities during initiation, RIP binds to and inhibits transcription elongation complexes (TEC) without displacing the nucleic acid scaffold from the TEC. RIP efficiently inhibits transcription directed from host as well as virus promoters, as a result the in vivo expression of RIP is extremely toxic to *Sulfolobus acidocaldarius* cells. The biological premise underlying a global shutdown of transcription is still unclear while a likely explanation is the evasion of host defence responses. RIP is a relatively abundant component of the ATV virion, which implies that transcription would already be repressed in the early stages of virus entry and infection prior to the formation of the viral lysogen, likely preventing the fateful activation of the host type III-B CRISPR system that is triggered by actively ongoing transcription. RIP is currently the only known archaeo-viral transcription regulator that directly targets the RNAP. The search for other archaeo-viral RNAP-binding transcription regulators has unearthed homologs not only in related viruses (SMV, STSV and ATSV) but also a paralog in ATV itself, the major coat protein ATV131, suggesting that RIP may be a representative of a novel family of transcription regulators. This family of proteins can be phylogenetically divided into two main clades depending on their similarity to either RIP or ATV131. A high-confidence structural homology model reveals RIP as a 4-helix bundle structure followed by a flexible C-terminal tail that is essential for RNAP-binding and function (Fig. 1f). The helical core of the proteins is highly conserved between the homologs whereas the tail extension differs significantly, implying that it is probably responsible for conferring specific functional properties.

## Summary and perspectives

The majority of transcription regulators encoded by archaeal viruses are repressors and many of them control their own expression (i.e. autoregulation). In addition to regulators encoded by the virus, it is noteworthy that regulators encoded by the cellular genome such as Sta1 have been implicated in the regulation of viral transcription (Kessler et al. 2006). While the structural characterization of transcription factors is well underway, their functional characterization in vivo is limited by the genetic tractability of archaeal viruses. In that respect, the team of Ken Stedman has carried out ground-breaking work by developing genetic tools in the *Sulfolobus* spindle-shaped virus 1 (SSV1) system (Iverson and Stedman 2012). Nearly half of the SSV1 genes were not required for virus function, revealing a significant correlation between the sequence conservation of a given gene across the *Fuselloviridae* family and its essential role for virus viability (Iverson et al. 2017). Importantly, the highly conserved (predicted) DNA-binding transcription factor ORFb129 is among the essential virus genes, while the poorly conserved (predicted) transcription factor VP2 is dispensable (Iverson and Stedman 2012). Our understanding of the regulatory logic underpinning viral transcription factors in archaea still leaves much room for improvement. F55, one of the more well-studied archaeo-viral regulators, seems to follow a regulatory strategy akin to the bacteriophage lambda CI repressor and plays a pivotal role in the lysogenic–lytic transition of the virus, and the expression of the early viral genes (Fusco et al. 2015). Overall, the DNA-binding, gene-specific regulators serve as molecular switches that ensure the correct temporal expression of genes that are required at distinct stages in the virus development.

Hitherto only a single global regulator encoded by archaeal viruses has been described: RIP which efficiently inhibits RNAP transcription of all genes, host and virus alike (Sheppard et al. 2016). The molecular mechanism of RIP is highly reminiscent of phage-encoded transcription regulators such as T7 Gp2 (Sheppard et al. 2013). T7 Gp2 is a potent repressor of the *E. coli* RNAP that enables the switch from host-RNAP transcription of host genes to bacteriophage-RNAP (T7) transcription of phage genes (Sheppard et al. 2013). However, unlike phage none of the archaeo-viruses apparently encode their own RNAPs. To date it is less clear how this regulatory strategy enables and orchestrates the viral gene expression programme, but it is likely that inhibition is a quantitative effect, more like a transcriptome attenuation than a global shut-down of RNA synthesis. In addition, it is possible that ATV encodes further regulators which are able to sequester RIP and negate inhibition on specific subsets of genes, possibly acting like anti-toxins. Sequence analysis of the ATV genome has revealed the putative DNA-binding proteins including ORF189 and ORF60 that both contain RHH domains, as well as a Zn-finger motif suggestive of DNA-binding transcription factors. The archaeo-viral transcription factors share similarities with both their bacterial and eukaryotic counterparts, and it is intriguing how an essentially eukaryote-like transcription system can be regulated by bacterial-like regulators. Whereas RHH proteins are almost entirely absent in eukaryotes but extremely prevalent in bacteria, the reverse is true for zinc-finger proteins. Thus, archaeal viruses employ a structural context and mechanisms of regulation adopted from all three domains of life. The elucidation of the structure, function and evolution of viral regulators, and the role of transcription in the virus–host relationship and arms race ARE an open and active field of investigation.
